# A 3D biofabricated cutaneous squamous cell carcinoma tissue model with multi-channel confocal microscopy imaging biomarkers to quantify antitumor effects of chemotherapeutics in tissue

**DOI:** 10.18632/oncotarget.27570

**Published:** 2020-07-07

**Authors:** James R. Browning, Paige Derr, Kristy Derr, Nicole Doudican, Sam Michael, Samantha R. Lish, Nicholas A. Taylor, James G. Krueger, Marc Ferrer, John A. Carucci, Daniel S. Gareau

**Affiliations:** ^1^ Laboratory for Investigative Dermatology, The Rockefeller University, New York, New York, USA; ^2^ National Center for Advancing Translational Sciences, National Institutes of Health, Rockville, Maryland, USA; ^3^ The Ronald O. Perelman Department of Dermatology, New York University School of Medicine, New York, New York, USA

**Keywords:** squamous cell carcinoma, screening, 3D printing, in vitro model, confocal microscopy

## Abstract

Cutaneous squamous cell carcinoma (cSCC) causes approximately 10,000 deaths annually in the U. S. Current therapies are largely ineffective against metastatic and locally advanced cSCC. There is a need to identify novel, effective, and less toxic small molecule cSCC therapeutics. We developed a 3-dimensional bioprinted skin (3DBPS) model of cSCC tumors together with a microscopy assay to test chemotherapeutic effects in tissue. The full thickness SCC tissue model was validated using hematoxylin and eosin (H&E) and immunohistochemical histological staining, confocal microscopy, and cDNA microarray analysis. A nondestructive, 3D fluorescence confocal imaging assay with tdTomato-labeled A431 SCC and ZsGreen-labeled keratinocytes was developed to test efficacy and general toxicity of chemotherapeutics. Fluorescence-derived imaging biomarkers indicated that 50% of cancer cells were killed in the tissue after 1μM 5-Fluorouracil 48-hour treatment, compared to a baseline of 12% for untreated controls. The imaging biomarkers also showed that normal keratinocytes were less affected by treatment (11% killed) than the untreated tissue, which had no significant killing effect. Data showed that 5-Fluorouracil selectively killed cSCC cells more than keratinocytes. Our 3DBPS assay platform provides cellular-level measurement of cell viability and can be adapted to achieve nondestructive high-throughput screening (HTS) in bio-fabricated tissues.

**Figure:**
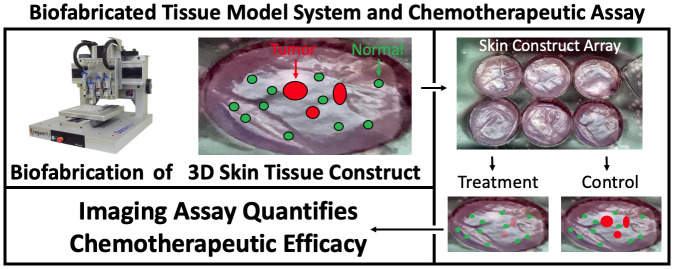


## INTRODUCTION

Global incidence of cSCC is 2.2 million people [1, 2] and accounts for most of the ~10,000 annual non-melanoma skin cancer deaths in the United States [3, 4]. cSCC treatments include excision, radiation therapy, photodynamic therapy, and/or topical treatment, including 5-fluorouracil (5FU) [5–7]. Drug discovery for small molecule therapies to treat locally advanced/inoperable or metastatic cSCC and other cancers can be accelerated using patient-specific, physiologically relevant models amenable to high-throughput screening. Models should mimic the tumor microenvironment, given its influence on tumor progression and metastasis [8], and should reproduce *in vivo* tumor cell physiochemical signaling and mechanical cues from the surrounding tissue extracellular matrix [9]. Animal models may not be readily translatable to human cancer treatment [10], and three dimensional (3D) tissue culture models offer a viable alternative for pre-clinical screening of small molecule therapeutics. 3D models using human derived cell lines offer increased complexity and physiological fidelity compared with two dimensional (2D) monocultures [11] and have been developed for several cancer models, including melanoma, pancreatic cancer, and cervical cancer [12–15]. Recently a human glioblastoma-on-a-chip model was described as more accurately capturing the tumor microenvironment and predicting patient-specific therapeutic response, as compared to 2D monoculture [16]. 2D and spheroid-only systems are unusable for the prediction of changes at the epithelial mesenchymal transition unlike the 3D system which can reflect important interactions between SCC, therapeutic molecules, and a collagen matrix. 2D cultures and spheroid models will not demonstrate collagen invasion and/or degradation and the diffusion limited size of an SCC spheroid with central necrosis is not a factor in monolayer cultures. 3D systems more accurately model diffusion limits of nutrients causing potential micro-metastases that have not yet acquired a vascular supply. In addition, the use of human specific 3D models has the potential for scalability, standardization and adaptability to high-throughput assay techniques.

**Figure 1:**
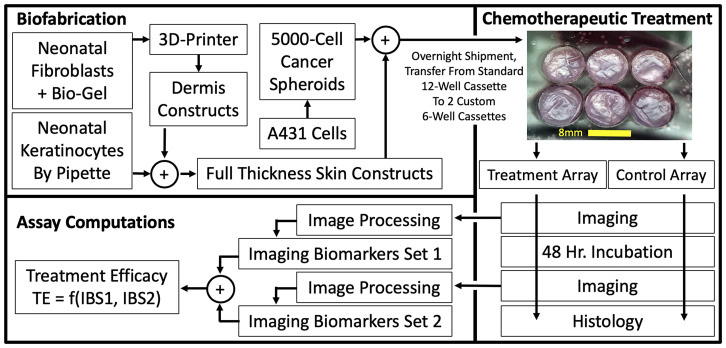
3D, Biofabricated tissue model system and chemotherapeutic assay using multi-channel confocal microscopy imaging biomarkers. From aliquots of cryopreserved cells, full thickness skin samples were biofabricated that mimic normal human skin morphology *in vivo*. After 4 days of media submersion, cancer spheroids were introduced that displaced and invaded the normal skin constructs. Constructs were transferred to our custom transwell apparatus to form construct arrays. Array cassettes were compatible with both incubation and imaging so they were imaged, treated by addition of 1μM 5FU to their nutrient media and re-imaged after incubation.

Engineered 3D skin tissue models produced using biofabrication techniques have been described [17, 18]. These tissues (Figure 1) are made with hydrogel scaffolding for dermal fibroblasts co-cultured with a surface layer of keratinocytes, and result in a 3D bi-layer model with structural similarities to human skin [19]. 3DBPS achieves precise architectures and better cellular placement than hydrogel models and can provide high-throughput, reproducible specimens [20]. It includes a 3D-printed fibroblast embedded collagen-based dermis and an epidermal layer of normal keratinocytes. The protocols for the 3-D printed fibroblast are also versatile and allow users, to readily introduce features of diseases. In the disease model presented here, A431 cSCC spheroids was introduced into the tissue, and histopathology and cDNA microarray analysis were used to confirm the biological fidelity of the cancer model.

Imaging biomarkers are quantifiable features of microscopy images that correlate with underlaying physiological processes of interest. The imaging/image processing assay computed imaging biomarkers (number of fluorescent cells and total amount of fluorescence) to define differential values before and after treatment that specified chemotherapeutic treatment efficacy, demonstrating different treatment effects on A431 cSCC cells versus normal (neonatal) keratinocytes.

Our objective was to quantify the therapeutic efficacy of a standard of care treatment for an cSCC skin tissue model that recapitulates the microenvironment in which this cancer grows. The 3D skin tissue cSCC assay was both scalable using current HT plate readers (i.e. fluorescence signal summation) and analytic on a cellular segmentation level, which may be more reliable due to optical turbidity in tissues [21]. Reflectance confocal microscopy (RCM) has been used for volumetric, *in vivo* imaging. In this protocol, multimodal confocal microscopy assayed our model system by tracking fluorescent cell populations, within reflective constructs, to quantify viability of normal and diseased tissue before and after chemotherapeutic treatment. Here we present a high-throughput-compatible, 3D-biofabricated cSCC skin tissue model system for efficacy testing of chemotherapeutics.

## RESULTS

**Figure 2:**
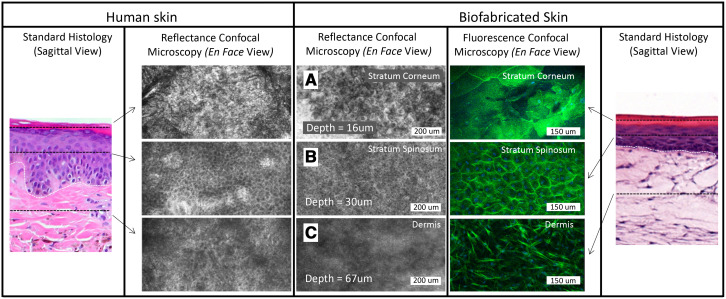
Stratum corneum (**A**), stratum spinosum (**B**), and dermis (**C**) of human skin and biofabricated skin at different depth layers in *en face,* confocal reflectance and fluorescence microscopy. The dermal-epidermal junction is shown as a dotted white line in the standard histology of skin and biofabricated skin images.

A complete protocol for *in vitro* therapeutic screening was developed using 3DBPS cSCC construct with dermal and epidermal layers biofabricated in 12-well transwell plates using a modification of a previously described technique [18]. Tissues were fabricated with A431 cSCC cells transduced with tdTomato red fluorescent protein (tdT-RFP) and 1% of normal primary keratinocyte transduced ZsGreen green fluorescent protein (Zs-GFP) were spiked into the epidermis as an internal control for non-desired toxic effects of the treatments. RCM confirmed morphological similarities between human and biofabricated skin (Figure 2).

**Figure 3:**
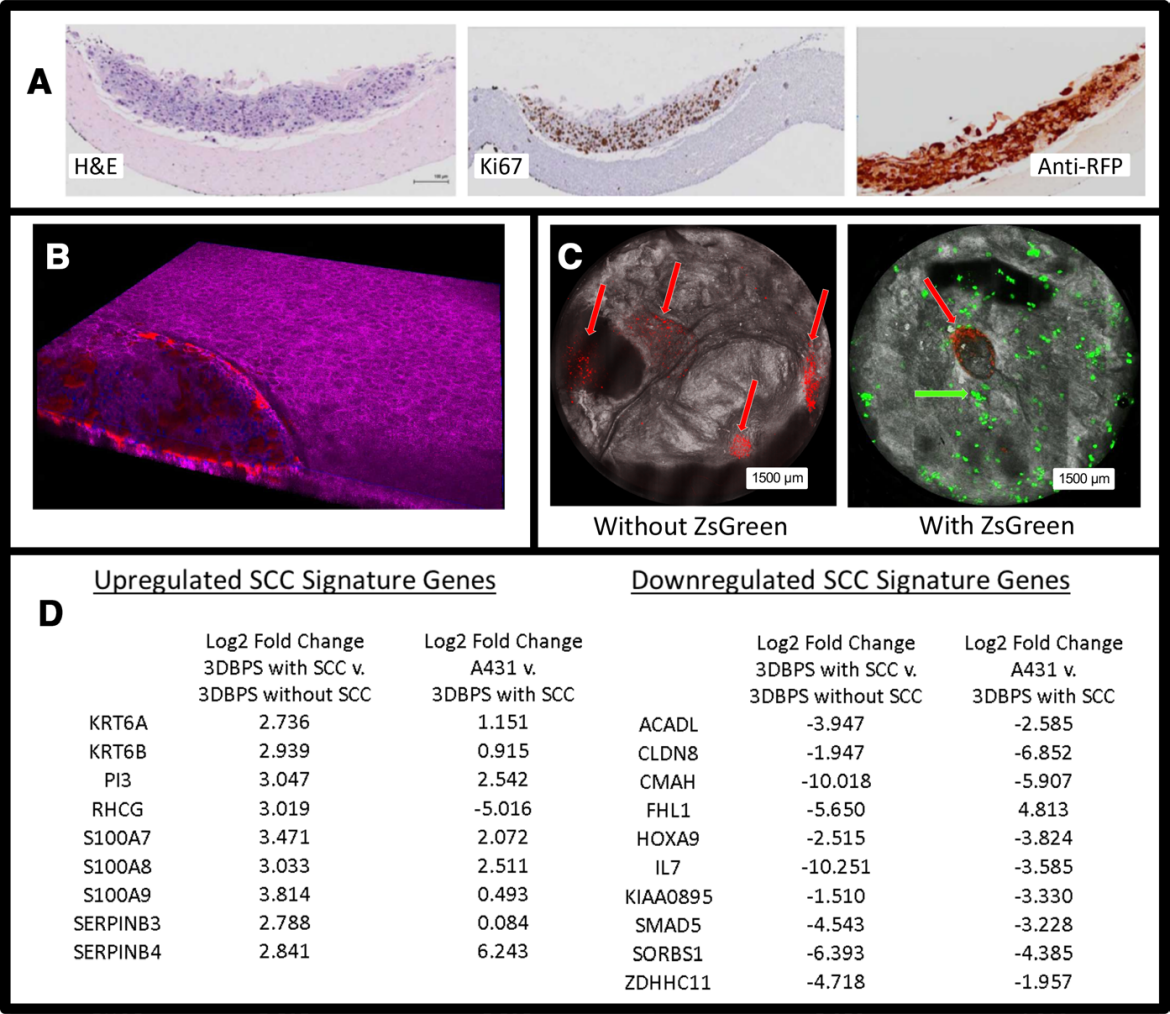
(**A**) H&E, Ki67, and Anti-RFP stained histology of 3DBPS model. (**B**) 3D confocal image of skin tissue with SCC tumor (purple: phalloidin staining, red: tdTomato red fluorescent protein (RFP) labeled SCC, blue: Hoescht staining). (**C**) Bimodal *en face* confocal microscopy of tdT-RFP-labeled cSCC (red arrows) in 3DBPS constructs with and without Zs-GFP-labeled neonatal keratinocytes (green arrow). All cSCC A431 cells are labeled with RFP while only 1% of keratinocytes are labeled with Zs-GFP resulting in sparsity of the green signal. (**D**) SCC signature genes that are up- and down-regulated in A431 cells grown *in vitro* and untreated full thickness 3DBPS without SCC spheroids compared to 3DBPS with SCC spheroids. Log2 fold change is normalized to Keratin 10.

*En face* RCM of a typical skin model biofabricated during this study showed a stratified model with well-formed stratum corneum, stratum spinosum, and dermis (Figure 2A–2C). The superficial stratified layer is 5–10 μm thick, with reflective cells under RCM. This is followed by a ~50 μm-thick layer of repeating honeycomb patterned granular dark nuclei cells resembling the stratum spinosum of human skin [18, 24]. RCM of the dermis is similar to that of human papillary dermis, but lacks the papillary network [24]. Fluorescence confocal microscopy (1.08 μm lateral resolution, 1.52–5.33 μm slice separation) confirmed integration of the A431 cells into the tissues, which produced a disruption of epidermal stratification in an otherwise well-differentiated epidermal layer (Figure 3A, 3B), by visualizing tdT-RFP while Zs-GFP labeled 1% of the keratinocytes were spiked into the epidermal layer (Figure 3C).

**Figure 4:**
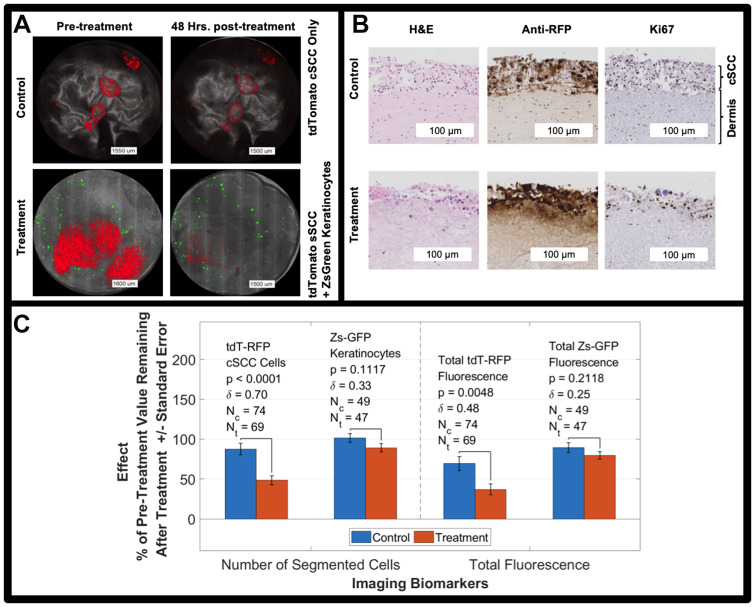
(**A**) Bimodal imaging examples of control and treated tumors (red) before and after the treatment period. cSCC tumors are red, reflectance is grayscale, and keratinocytes are green. All cSCC A431 cells are labeled with RFP while only 1% of keratinocytes are labeled with Zs-GFP resulting in sparsity of the green signal. (**B**) H&E, Ki67, and Anti-RFP antibody staining of untreated (control) tumor model and tumor model treated with 5FU for 48 hours. (**C**) 5FU treatment effect on tdT-RFP-labeled cSCC spheroids and Zs-GFP-labeled keratinocytes including *p*-value and effect size, *δ*. Two imaging biomarkers are shown, number of segmented objects and total fluorescence of the size-thresholded segmented objects. NOTE: the fluorescence in panel A is intentionally saturated for display.

Both Zs-GFP and tdT-RFP images were segmented, enabling automatic, volumetric cell counting. We formulated the first imaging biomarker as the number of cells in the 3D tissue. Our second imaging biomarker was the total Zs-GFP fluorescence signal within each construct and total tdT-RFP florescence signal within each tumor spheroid. RNA-Seq analysis on 3DBPS-SCC (A431) vs. 3DBPS alone and 3DBPS-SCC (A431) vs. A431 assessed model integrity at the level of gene expression, showing increased expression in 3DBPS-SCC which corresponds to our previous finding of increased expression at the leading edge of invasive human cSCC *in vivo* [25]. These included S100A7, S100A8, S100A9, KRT6A, SERPINB3, SERPINB4, and PI3. Gene expression was normalized to keratin 10 for increased specificity to cSCC vs. increased numbers of activated keratinocytes. Downregulated genes included IL-7 which was also downregulated at the leading edge of invasive cSCC *in vivo* (Figure 3D). We observed that tumor cells proliferated into the surrounding epidermis prior to 5FU treatment and formed a crater in the epidermis. To quantify therapeutic effect, we calculated a ratio of tumor fluorescence signal at the end of the treatment period between wells with mock treatment and those in which the drug was added. In 5FU-treated tumors, tdT-RFP fluorescence was reduced after treatment, especially in some tumor cores (Figure 4A). Ki67 staining indicated greater cSCC proliferation in controls compared to treated samples, which had fewer Ki67+ nuclei. Anti-RFP immunohistochemistry showed thicker untreated tumors with ~10 layers of cells compared to ~5 layers for treated tumors (Figure 4B).

We counted cells by fluorescence segmentation as our primary imaging biomarker before and after the 5FU treatment period [1] for samples fed by 5FU-treated media versus control samples fed by pure media and [2] for tdT-RFP cSCC cells and Zs-GFP using different fluorescence imaging channels. Table 1 shows our results. A two-way ANOVA for unbalanced design with cell type (A431 cSCC vs. keratinocytes) and treatment (untreated vs. treated) as factors resulted in a cell type-treatment interaction *p*-value of 0.039 indicating that 5FU differentially changed outcome for cSCC versus keratinocytes. A dose-response study showed increased reduction in the number of segmented cells for a 2 μM 5FU treatment compared to untreated controls versus a 1 μM treatment compared to untreated controls: 71.8 ± 47.2%, *n* = 15; 52.0 ± 41.3%, *n* = 18; and 34.7±36.1%, n = 20, respectively (Supplementary Figure 1).

**Table 1:** Imaging biomarker values: number of cells and total fluorescence as a post treatment percentage of their pre-treatment values

	Control Group		Treatment Group			
	Mean±SD	*N*	Mean ± SD2	*N* 3	*P*-value	Hedges g
%tdT-RFP cells	87 ± 62	74	48 ± 46	69	4.29E-05	0.703
%Zs-GFP cells	101 ± 38	49	89 ± 36	47	0.1117	0.3251
%Total tDT-RFP	69 ± 75	74	37 ± 57	69	0.0048	0.4773
%Total Zs-GFP	89 ± 42	49	79 ± 31	47	0.2118	0.2546

The primary fluorescence imaging biomarker quantifying the percent of pre-treatment tdT-RFP-labeled cells remaining after treatment showed significant decreases in 5FU-treated tumors versus untreated controls. For Zs-GFP keratinocytes, correlating differences were not significant. There was a significant difference in treatment effect measured by the secondary imaging biomarker (total fluorescence) for tdT-RFP cSCC spheroids between treated samples and untreated controls.

## DISCUSSION

The present studies are remarkable for the following: (1) we developed and validated a morphologically and genomically accurate 3D skin cSCC tissue model that enables study of the effect of chemotherapeutics on cancer cells growing in the context of their native tissue microenvironment; (2) we developed a nondestructive, 3D fluorescence confocal imaging assay to test both efficacy and general toxicity of anti-cancer chemotherapeutics; (3) we derived imaging biomarkers to quantify therapeutic effect in our system; and (4) we pharmacologically validated our assay by demonstrating different effects of 5-flurouracil on A431 SCC cells versus normal keratinocytes in the 3D model system.

Thereare currently several approaches to test the cytotoxic effects ofchemotherapeutics on cancer cells *in vitro*, from 2D monolayer systems to patient-derived organoids. Our novel3DBPS cSCC model provides several advantages over current methods: (1) it provides a relevant tissue context to the growth of cSCC cancer cells; (2) it enables non-destructive cellular monitoring during drug therapy on cSCC growth within skin tissue; (3) it recapitulates native cSCC biology, exhibiting similar histology and gene expression to invasive cSCC *in vivo*; and (4) it can be adapted as a HTS platform for evaluation of small molecule therapy intreatment of locally advanced, inoperable or metastatic cSCC. The 3DBPS model has been validated on morphological, molecular, and functional bases through investigation of pathology and confocal imaging, gene expression, and response to treatment respectively.

The model performed exceptionally well on the basis of morphology as determined by light and confocal microscopy. H&E evaluation of 3DBPS demonstrated a collagen-rich dermis with proliferative fibroblasts and a confluent layer of basal keratinocytes. Although SCC was seeded as spheroids within the 3DBPS, it exhibited vertical and radial growth. H&E, Ki67, and anti-RFP staining of treated and untreated 3DBPS cSCC skin models illustrated pharmacological effects after 48-hour 1 μM 5FU treatment, including decreased cSCC cells, and more compact architecture of tumor cells in the treatment group. Bimodal confocal visualization showed a proliferating perimeter of cSCC cells, often surrounding a non-fluorescent tumor core visualized by RCM. This suggested central necrosis. Prior to treatment, cSCC spheroids proliferated into and disrupted the normal epidermis. cSCC spheroids typically caused craters in the epidermis and extended into the dermis.

*En face* confocal imaging showed that 3DBPS resembled human skin, with three layers: stratum corneum, stratum spinosum, and dermis. The stratum spinosum of the 3DBPS showed the dark nuclei honeycomb pattern of keratinocytes which transitioned into larger, flatter, bright nuclei cells in the stratum corneum, indicating differentiation of a stratified epithelium consistent with human skin and high-level structural fidelity of the model.

We evaluated the model at the level of gene expression using RNA-Seq analysis, demonstrating increased expression of genes upregulated in cSCC *in vivo*, including keratin self-signaling genes. We identified eight genes that showed increased expression in spheroids that overlapped with genes we isolated at the leading edge of cSCC including S100A7, S100A8, S100A9, KRT6A, SERPINB3, SERPINB4, PI3, and RHCG. Of note, SERPINB3 (SCC antigen) was highly expressed in 3DBPS cSCC spheroids while showing low expression for 3DBPS without cSCC. SERPINB3 expression by 3DBPS with cSCC approached levels observed in A431 cells, supporting fidelity of our model at the gene expression level. SERPINB3 is highly expressed in other hyper-proliferative skin disorders, particularly psoriasis [26]. S100A7, which was expressed highly by 3DBPS with cSCC, is known to promote migration, invasion, and metastasis of cervical cancer and is highly expressed in human cSCC [25, 26]. S100A8/A9 form a calcium binding heterodimer that plays a role in leukocyte recruitment and myeloid cell function. KRT6A is highly expressed in aggressive SCC of the lung and leading edge of cSCC invadopodia [25, 27]. SERPINB3 (cSCC antigen) and B4 are highly expressed in cSCC and benign hyperproliferation (i. e., psoriasis). Rh family glycoproteins are highly expressed in SCC of the head and neck (SCCHN), are poor prognostic indicators for lung cancer [28]. Expression of these genes by A431 spheroids in the 3DBPS cSCC model demonstrates consistency with gene expression observed at the leading edge of invasive cSCC. Downregulation of IL-7 is consistent with blunting of immune response, particularly T cell immunity at leading edge of invasive carcinoma.

Our assay and model system were evaluated using imaging biomarkers. We evaluated functional performance of the model via total tumor fluorescence and number of cells, where the total fluorescence was quantified as the sum of the fluorescent intensity over the entire 3D confocal fluorescence image of the construct. We saw the same trend for both imaging biomarkers: quantified values decreased with treatment when computed using the red fluorescence from the tumor cells and did not change the Zs-GFP keratinocytes in a statistically significant way.

2-way ANOVA showed that that 5FU differentially changed the outcome for cSCC versus keratinocytes indicating selective killing of cSCC cells. The response to 5FU was 50% decrease in tumor cells post-treatment compared to <10% for untreated controls. Zs-GFP normal keratinocytes increased in untreated control samples and decreased in treated samples, although this trend wasn’t significant (*p =* 0.0553). Increasing dose of 5FU for a smaller set of samples (N_control_ = 15, N_1× dose_ = 18, N_2 × dose_ = 20) showed an expected increase in treatment effect. This novel 3DBPS cSCC model can be used for non-destructive efficacy assessment of candidate therapies, more closely recapitulates invasive cSCC biology than current methods, and can be adapted for HTS of small molecule therapy for cSCC.

The model described provides a higher degree of clinical relevance because it enables the testing of chemotherapeutics against tumor cell growth in a tissue specific context, thus capturing any potential interactions between the tumor and its microenvironment. We envision that this model could be adopted in a “bedside” manner and applied to cells from cSCC patient tumor biopsies.

## MATERIALS AND METHODS

### Cell culture

Neonatal human dermal fibroblasts (HDFN, Zen Bio DFN-F) were cultured at 37° C, 5% CO_2_ in Dulbecco’s Modified Eagle’s Medium (Gibco 11965), 10% fetal bovine serum (Hyclone), and 1% penicillin-streptomycin (Gibco). Neonatal keratinocytes (NHEKN, ScienCell 2100) were cultured at 37° C, 5% CO_2_ in Keratinocyte Media (Lonza 192060). SCC A431cells (ATCC^®^ CRL-1555) were cultured at 37° C, 5% CO_2_ in Dulbecco’s Modified Eagle’s Medium (Gibco 11965), 10% fetal bovine serum (Hyclone), and 1% penicillin-streptomycin (Gibco). NHEKN and A431 were transduced with Zs-GFP and tdT-RFP lentiviral vectors (Vectalys 0010VCT, 0008VCT), respectively, following the vendor provided protocol (MOI = 10, 4 μg/mL polybrene, 4-hour incubation at 37° C).

### Biofabricated cSCC skin models

Skin models were biofabricated in 12-well transwell plates as described previously [18] with the only modification being that the basement membrane layer (Laminin/Entactin solution) and the NHEK_N_ cell suspension were applied by hand pipetting. Zs-GFP-expressing NHEK_N_ were mixed homogenously with non-fluorescent NHEK_N_ at 1% prior to application of the cell suspension. After 1.5-hour room temperature incubation, tissues were submerged and incubated at 37° C. On day three after tissues were printed, tdT-RFP-expressing A431 cSCC cells were pipetted at 5000 cells/well in 50 μL into a 384-well low-attachment plate (Nexcelom ULA-3840-020) to promote spheroid formation. On day four, A431 spheroids were pipetted onto the top surface of tissue (five spheroids per tissue). Tissues were subsequently incubated for three more days submerged and seven days with the air-liquid interface as described [18].

### Histology

3DBPS samples were bisected and fixed in 10% neutral formalin for 72 hours, transferred to 70% ethanol and processed to paraffin. Scaffolds were embedded with the bisected surface down, collected at 4μm on Plus slides (Fisher Scientific, Cat # 22-042-924) and stored at room temperature prior to use. One section was stained with standard H&E. Chromogenic immunohistochemistry was performed on a Ventana Medical Systems Discovery XT instrument with online deparaffinization using Ventana’s reagents and detection kits unless otherwise noted (Ventana-Roche Diagnostics, Indianapolis IN, USA). tdTomato-expressing cells were detected with polyclonal rabbit anti-RFP (Rockland Cat# 600-401-379 Lot# 35055 RRID: AB_2335885) raised against a fusion protein corresponding to the full-length amino acid sequence from mushroom coral Discosoma. Proliferating cells were detected with rabbit anti-mouse Ki67, clone SP6 (Spring Biosciences Cat# M3062 Lot# 160726LVS RRID: AB_11219741).

Sections were incubated for 1 hour at 60° C followed by online deparaffinization. tdTomato was antigen-retrieved using protease-3 (Ventana Medical Systems) for 12 minutes. Ki67 was heat retrieved for 36 minutes in Ventana Cell Conditioner 1 (Tris-Borate-EDTA pH 8.5). Endogenous peroxidase activity was blocked with hydrogen peroxide. tdTomato was diluted 1:1600 in Tris-BSA (25 mM Tris, 15 mM NaCL, 1% BSA, pH 7.2) and incubated for 12 hours at room temperature. Ki67 was diluted 1:400 in Ventana Antibody Dilution Buffer (Catalog# 251-018) and incubated for 1 hour at 37° C. tdTomato was detected with biotinylated, goat anti-rabbit (Vector Laboratories Cat#Ba-1000 Lot#ZA0324 RRID: AB_2313606) diluted 1:200 in Tris-BSA and incubated for 30 minutes at 37° C. This was followed by the application of streptavidin-horseradish-peroxidase conjugate. Ki67 was detected with goat anti-rabbit horseradish peroxidase conjugated multimer and incubated for eight minutes. Both antibodies were visualized with 3,3′-diaminobenzidene and enhanced with copper sulfate. Slides were washed in distilled water followed by counterstaining with hematoxylin, dehydrated, and mounted with permanent media. Appropriate positive and negative controls were run with study samples. These procedures were performed by the NYU Center for Biospecimen Research and Development (CBRD) core facility.

### cDNA gene profiling

RNA was prepared from samples using RNeasy Mini Kit (Qiagen). RNA quality was assessed on the Agilent Bioanalyzer 2100 platform (Agilent, Santa Clara, CA). Libraries were prepared with the TruSeq RNA Sample Preparation Kit (Illumina). Sequencing (RNA-Seq) was done with the Illumina Next-Gen Sequencing HiSeq (Illumina, San Diego, CA, USA) with 30–45 million 50-bp, paired-end reads at the NYU Genome Technology Core. RNA-Seq raw reads were normalized using STAR software. Differential expression data were obtained using the DEseq algorithm. Analyses were completed with the Basepair (New York, NY) software.

### Sample preparation

Samples were transferred (12 samples in a 12-well cassette, Product 3460, Corning Life Sciences, Corning NY, USA) via overnight courier with a cool pack in a polystyrene foam container from the National Center for Advancing Translational Sciences site to The Rockefeller University for processing, treatment, and imaging. Samples were separated into control and treatment groups and visually examined to guide an 8-mm punch biopsy to the area appearing to contain the most uniform epidermis and highest number of tumors, which typically occupied the central half of the 12-mm diameter. A fresh biopsy punch was used every three samples. The 8mm punches shrunk slightly so we LASER-cut circular wells (7.60-mm diameter = 7.50 mm cutting pattern +0.10 mm dilation due to cutting line width) in a circular slab of 1.02-mm thick acrylic (Product ACRYCLR0.040PM12 × 36, ePlastics, San Diego, CA, USA) to insert into a 75-mm transwell (Product 3419, Corning Life Sciences, Corning, NY, USA) to form wells (Figure 1). Supplementary Figure 2 demonstrates the optical coupling to submerge samples just beneath cover glass in the LASER-cut well structure for a 3-mm construct version that we test prepped to investigate HT compatibility in the 384-well format. Both the 8-mm and 3-mm custom well structures allowed sample preparation that is compatible with printing, incubation and imaging. Ki67 histological analysis showed that untreated cSCC cells from control samples were proliferative after two imaging sessions.

### Confocal imaging

Reflectance and fluorescence confocal 3D images were collected under 488nm and 532 nm LASER excitation for a total of 4 imaging modes using a 10× air-immersion lens (RS-G4, Caliber ID Inc., Rochester NY, USA retrofitted with 488- and 532-nm LASERs from Toptica Photonics Inc., Farmington NY, USA). Lateral pixel resolution was 1.1 μm, and stack spacing ranged from 3.1 to 5.3 μm. Confocal images (~500GB) were acquired for 2 × 3 arrays of 6 specimens (e. g., Figure 1) (24 mm × 16 mm × 0.5 mm). Imaging and analysis were performed on seventeen sequential sample batches, four of which were excluded based on the criteria that in these batches, an incorrect optical filter setting caused image bleed-through between the fluorescence channels of the microscope, misregistering keratinocytes as A431 cSCC cells.

### Image analysis

SCC cells (red fluorescence) and keratinocytes (green fluorescence) were overlaid in semi-opaque masks on grayscale RCM. Opacity of masks was linearly proportional to signal, and signal was multiplied by a constant for the purposes of visualization. Because tdT-RFP and Zs-GFP images were each acquired simultaneously with a corresponding RCM stack, the two fluorescent channels were spatially registered for rotation and translation using their respective z-sum RCM images and the function imregtform in MATLAB (MATLAB 2018b, The Mathworks, Natick, MA, USA). 3D fluorescent image stacks were segmented using custom software written in MATLAB (Supplementary Figure 3). Fluorescence signal for each 3D voxel was normalized for LASER power and detector (photomultiplier) gain to a standard image. LASER and detector settings were consistent between pre- and post-images for each sample but varied between sample batches.

Segmentation used a Gaussian smoothing filter with a kernel size of 8, binarization used Otsu’s method [22] and removal of connected components containing 4 or fewer voxels with 26-connectivity (i. e., adjacent corners, edges, and faces connect voxels). Image smoothing and small object elimination reduced background noise in images. Imaging biomarkers were calculated for entire samples in the case of Zs-GFP-labeled keratinocytes and for individual tumors for tdTomato-labeled SCC. The differences in pre- and post-imaging biomarkers between treated samples and untreated controls were compared using an unpaired two-sample *t*-test. Hedges’ g was calculated as a measure of effect size, *δ*, using the open source Measure of Effect Size Toolbox [23]. Treatment effect for keratinocytes and cSCC tumors was calculated as the post treatment percent of pre-treatment values for the imaging biomarkers (Table 1). Two-way ANOVA for unbalanced design with cell type (A431 cSCC vs. keratinocytes) and treatment (untreated vs. treated) as factors used the anovan function in MATLAB.

### 5-Fluorouracil treatment

The control and treatment groups were each seated in separate transwells, wherein 8 mL of media fed the constructs over the 48-hour treatment course. The control group contained pure media, while the treatment group contained media with 1 μM 5FU (Product F6627, Sigma Aldrich, St Louis, MO, USA). For three batches of samples, an additional treatment group was seated in a transwell assembly containing a double (2 μM 5FU) dose.

## SUPPLEMENTARY MATERIALS



## Abbreviations

cSCC: Cutaneous Squamous Cell Carcinoma
3DBPS: 3 Dimensional Bio-Printed Skin
H&E: Hematoxylin and Eosin
5FU: 5-Fluorouracil
Zs-GFP: Zs Green Fluorescent Protein
tdT-RFP: tdTomato Red fluorescent Protein
RCM: Reflectance Confocal Microscopy
HTS: High Throughput Screening
