# Supplementary material for: A 3D biofabricated cutaneous squamous cell carcinoma tissue model with multi-channel confocal microscopy imaging biomarkers to quantify antitumor effects of chemotherapeutics in tissue
Source: Oncotarget. 2020 Jul 7;11(27):2587–96. doi: 10.18632/oncotarget.27570 (PMC7343636; doi:10.18632/oncotarget.27570)
Supplement: Supplementary file 1 [file oncotarget-11-2587-s001.pdf]

A 3D biofabricated cutaneous squamous cell carcinoma tissue model with multi-channel confocal microscopy imaging biomarkers to quantify antitumor effects of chemotherapeutics in tissue

SUPPLEMENTARY MATERIALS

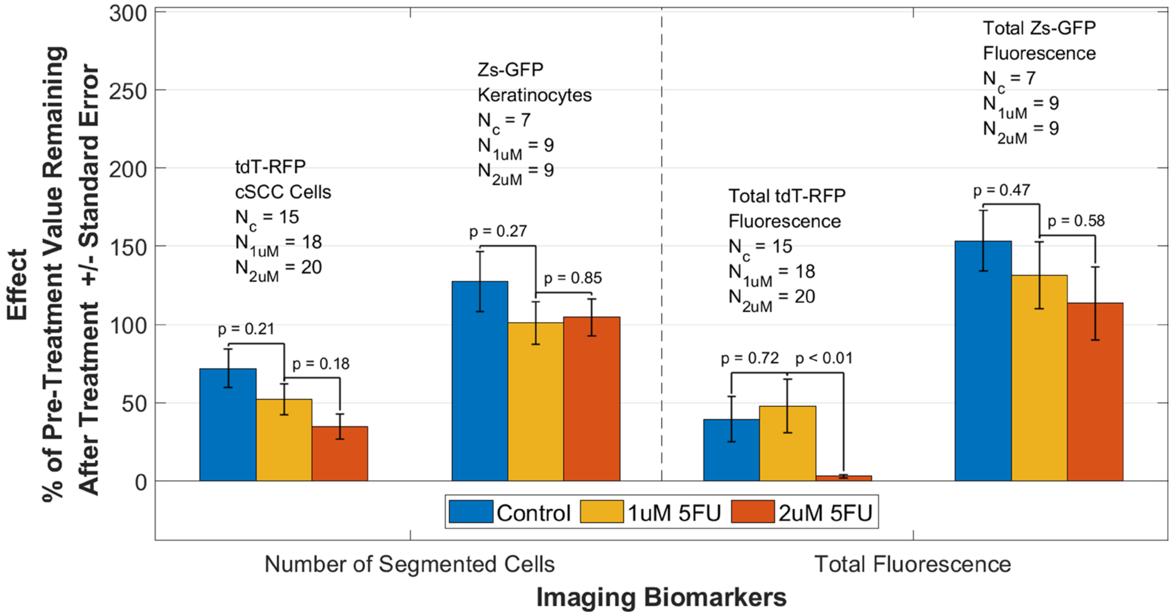

Supplementary Figure 1: 5FU treatment dose response on tdT-RFP-labeled cSCC spheroids and Zs-GFP-labeled keratinocytes.

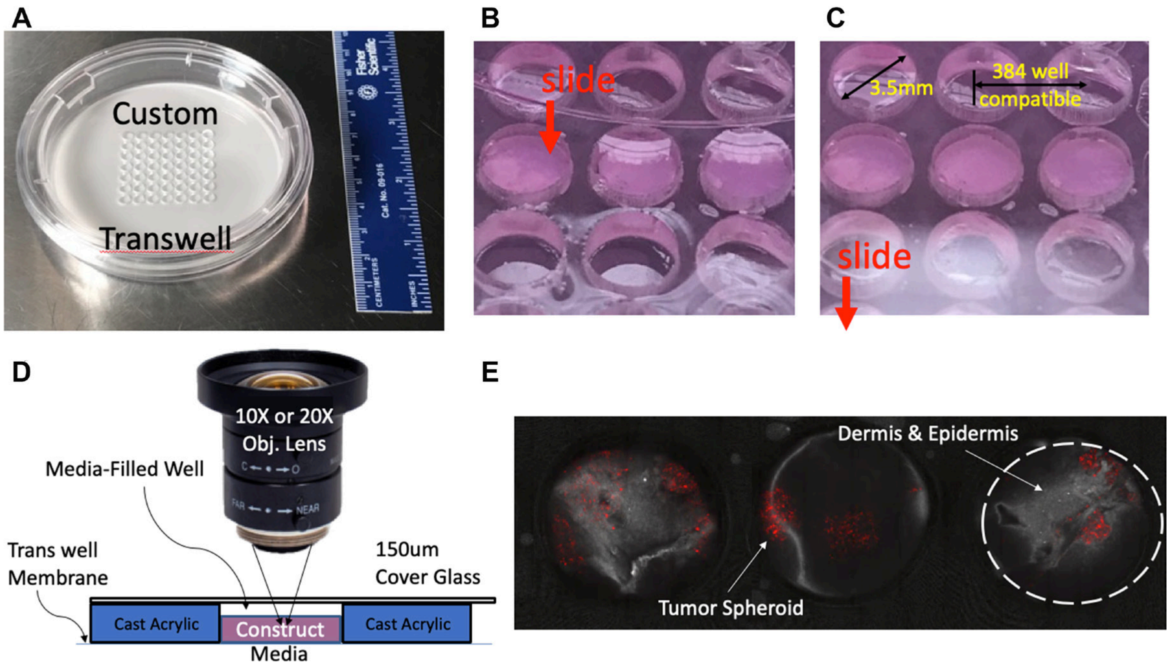

Supplementary Figure 2: (**A**) High-throughput compatible custom transwell device for incubation and imaging. (**B**) Bioprinted skin samples are placed in the wells, followed by additional media and a glass coverslip to form a glass-liquid interface above the skin. (**C**) The well array spacing is identical to the spacing of the pins in standard pin tools for compound delivery. The 40-mm round cover slip can slide (downward, red arrow) to provide immersing media coupling during confocal microscopy. (**D**) The transwell device is used for confocal imaging using a standard 10× or 20× objective lens, application of therapeutic dose, and for incubation allowing tissue samples to be imaged, treated, and incubated without removal from device. (**E**) Example of resulting bimodal confocal *en face* image showing epidermis in reflectance grayscale and tdT-RFP-labeled A431 cSCC cells in red.

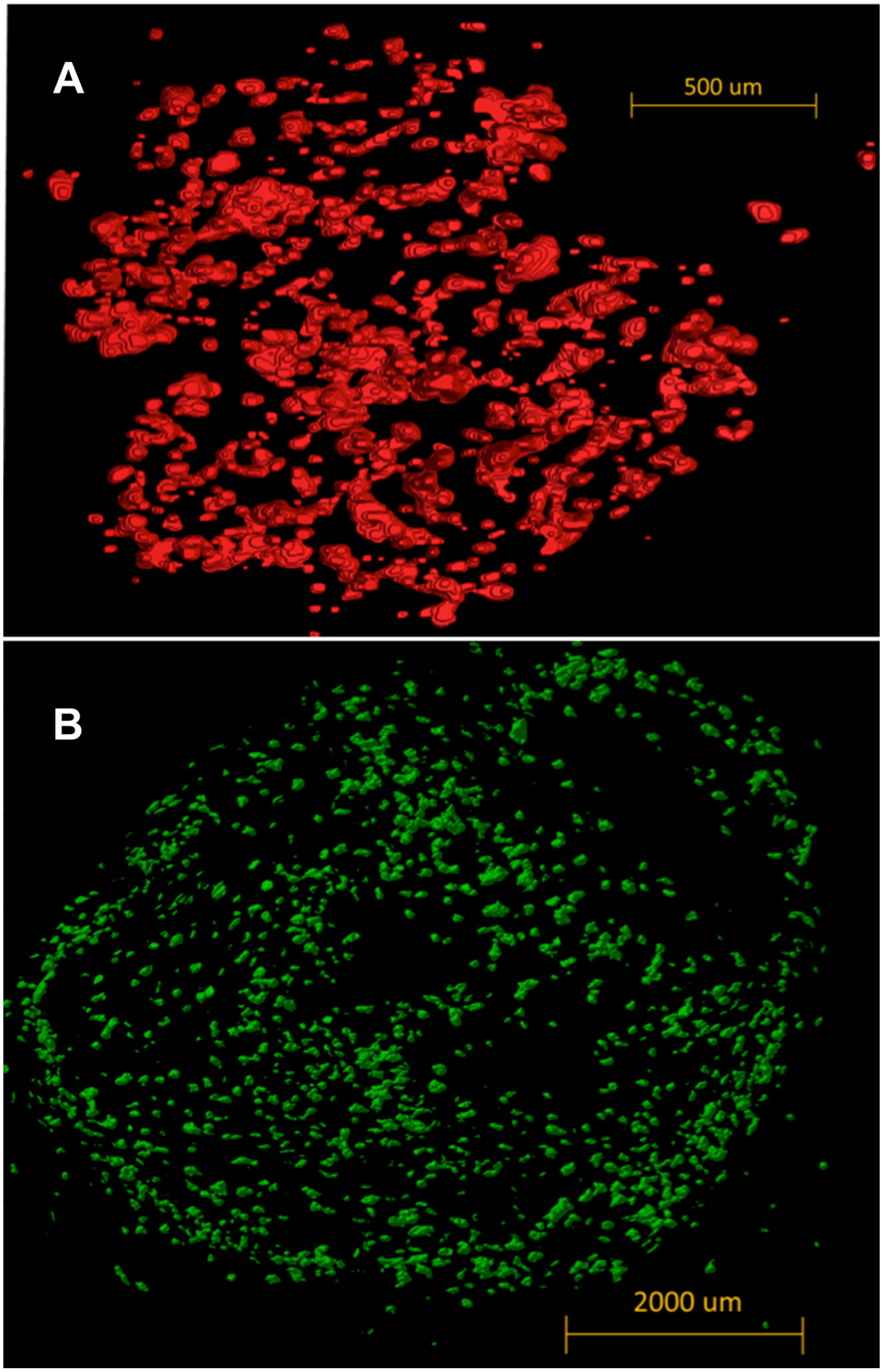

Supplementary Figure 3: Segmentation of cells for (**A**) tdT-RFP-labeled cSCC tumor spheroid and (**B**) Zs-GFP-labeled keratinocytes used to derive quantitative therapeutic effect imaging biomarkers. 1% of keratinocytes are labeled resulting in a sparse signal and improved separation of objects.
